# Statins Are Associated with Improved Survival of Patients with Gastric Cancer: A Systematic Review and Meta-Analysis

**DOI:** 10.1155/2022/4938539

**Published:** 2022-05-17

**Authors:** Mingjie Yuan, Shuyi Han, Yanfei Jia, Jiankai Feng, Duanrui Liu, Zhenguo Su, Xiangdong Liu

**Affiliations:** ^1^Department of Clinical Laboratory, Binzhou Medical University, Yantai, Shandong, China; ^2^Department of Laboratory, Jinan Central Hospital Affiliated to Shandong First Medical University, Jinan, Shandong, China; ^3^Department of Laboratory, Jinan Central Hospital Affiliated to Shandong University, Jinan, Shandong, China; ^4^Department of Clinical Laboratory, Shandong Provincial Hospital Affiliated to Shandong First Medical University, Jinan, Shandong, China

## Abstract

Statins are associated with gastric cancer (GC) risk. The present study aimed to clarify the efficacy of statins on the overall survival (OS) benefits in patients with GC. Publications were retrieved from PubMed, Embase, and the Cochrane Library as of April 2022. Data from the eligible cohort, case-control studies, and randomized control trials (RCTs) were extracted for the meta-analysis. Hazard ratio (HR) and 95% confidence intervals (CI) were used to assess the association between statins users and OS in GC patients. Subgroup analysis was performed based on the study design (prospective *vs*. retrospective). A total of 6 studies encompassing 5693 GC patients were included. Statins added to the standard treatment prolonged the patient's OS outcome (HR (95% CI): 0.72 (0.53–0.97), *p* = 0.032; *I*^2^ = 88.0%, *p*_*heterogeneity*_ < 0.001). A prospective study did not find any statistically significant difference in OS between statins users *vs*. nonstatin users (HR (95% CI): 0.92 (0.68–1.26), *p* = 0.614; *I*^2^ = 11.7%, *p*_*heterogeneity*_ = 0.322), whereas the retrospective studies showed prolonged OS in statins users (HR (95% CI): 0.63 (0.42–0.961), *p* = 0.032; *I*^2^ = 94.6%, *p*_*heterogeneity*_ < 0.001). Statin users had significantly improved OS compared to nonstatin users in GC treatment. This long-term survival benefit was only observed in the pooled analysis of retrospective studies but not in prospective studies.

## 1. Introduction

Gastric cancer (GC) is the fifth most common cancer worldwide, with an annual incidence of approximately 1 million, and the third leading cause of cancer deaths, accounting for >0.7 million deaths annually [1, 2]. The five-year survival rate for advanced GC is <30% [3–5]. Men are two to three times more likely to develop GC than women [5]. The regions such as Eastern and Central Asia and Central and South America have the highest incidence rate of GC [6]. The risk factors for GC include *Helicobacter pylori* infection, gender, age, ethnicity, family history, diet, alcohol consumption, and smoking [1, 2, 7].

Statins, the 3-hydroxy-3-methylglutaryl-coenzyme A (HMG-CoA) reductase inhibitors, are currently the most commonly prescribed pharmaceutical drugs worldwide [8, 9]. Statins exhibit a well-established efficacy in lowering blood cholesterol levels and reducing cardiovascular events and could be used for primary and secondary prevention in various patient populations [10–12]. Currently, seven statins are available in the market: atorvastatin [13], fluvastatin [14], lovastatin [15], pitavastatin [16], pravastatin [17], rosuvastatin [18], and simvastatin [19]. The primary mechanism of statins in reducing serum cholesterol levels is through competitive and reversible inhibition of HMG-CoA reductase, which is the rate-limiting step in the mevalonate synthesis pathway [20]. Statin therapy can also lower the level of oxidized low-density lipoprotein (ox-LDL), which is a major risk factor for atherosclerosis [21]. Moreover, the antitumor properties of statins have been demonstrated in GC cell lines *in vitro*, and the mechanism may involve inhibiting genes related to cell division, activating apoptosis, and suppressing YAP and *β*-catenin signaling [22–25]. MEK5/ERK5 knockdown sensitizes small-cell lung cancer (SCLC) cells to statin inhibition of the mevalonate pathway [26]. *In vivo* animal model studies showed that statin therapy prevents cancer development and growth [27–29]. Simvastatin can inhibit the HIF-1*α*/PPAR-*γ*/PKM2 axis and suppress PKM2-mediated glycolysis, resulting in decreased tumor cell proliferation and increased apoptosis in a xenograft hepatocellular carcinoma (HCC) mouse model [30]. Clinical evidence supported the protective effect of statins on reducing breast cancer recurrence [31]. Studies have also shown that statin usage can reduce cancer-specific mortality [32, 33].

Several clinical studies have shown that statins combined with radiotherapy or chemotherapy are associated with GC risk, but the results are still controversial [34–37]. A meta-analysis indicated that statin could cause a 32% reduction in GC risk [38]. However, the effect of statin use on the prognosis in patients with GC after treatments remains unclear [39–42]. Previous studies showed that statin had no impact on the long-term outcomes of progression-free survival (PFS) and overall survival (OS) in GC patients [43]. Recently published studies have demonstrated that statin combined with standard cancer treatment reduces the patient's mortality [39, 44]. Therefore, we conducted this systematic review and meta-analysis to update the related randomized controlled trials (RCTs) and observational studies and comprehensively investigated the effect of statin use on the long-term outcomes of GC patients' postsurgery and adjuvant chemotherapies.

## 2. Materials and Methods

According to the Preferred Reporting Items for Systematic Reviews and Meta-Analyses (PRISMA) guidelines, we conducted a systematic review and meta-analysis.

The relevant articles were searched using the PICOS principle, followed by screening based on the inclusion and exclusion criteria. The extracted data included baseline characteristics. The end-point data were reviewed by two investigators (Mingjie Yuan and Shuyi Han) according to the prespecified protocol.

### 2.1. Eligibility Criteria

The study inclusion criteria were as follows: (1) patients with histologically or cytologically confirmed stomach adenocarcinoma who received standard treatment (surgery and/or chemotherapy and/or radiotherapy); (2) statin treatment as study intervention, and statin users received at least one prescription of any statin (atorvastatin, fluvastatin, lovastatin, pitavastatin, pravastatin, rosuvastatin, and simvastatin); (3) studies assessing the association between statin use and patients' survival outcome using the hazards ratio (HR) with its 95% confidence interval (95% CI); (4) publication language was limited to English; and (5) relevant RCT, cohort, and case-control studies were included. The exclusion criteria were as follows: (1) reviews, conference abstracts, editorials, letters, meta-analyses, case reports, and experimental animal studies, (2) insufficient data, and (3) full text unavailable.

### 2.2. Search Strategy

PubMed, Embase, and Cochrane Library databases were searched up to April 2022 for potentially eligible studies. For the search, we used the MeSH terms, “hydroxymethylglutaryl-CoA reductase inhibitor(s),” “statin(s),” “fluvastatin,” “lovastatin,” “atorvastatin,” “pravastatin,” “rosuvastatin,” “pitavastatin,” “simvastatin,” combined with “stomach neoplasms” and “survival,” “prognosis,” or “death,” as well as relevant keywords. The language was limited to English.

### 2.3. Data Extraction

The following data were extracted from each study: authors, year of publication, the country where the study was performed, study design, sex (exposed to statins vs. not exposed), sample size (exposed to statins vs. not exposed), age (exposed to statins vs. not exposed), previous treatment, and follow-up duration. HR and its 95% CI of survival outcome with and without adjustments for potential confounders were also collected.

### 2.4. Quality of the Evidence

Herein, a total of six studies were included in our final model. The level of evidence of all articles was assessed independently by two authors (Mingjie Yuan and Shuyi Han) according to the Newcastle–Ottawa scale (NOS) criteria for quality assessment of cohort and case-control studies. In addition, we assessed the quality of RCT by using version 2 of the Cochrane risk-of-bias assessment tool. The discrepancy in the assessment was resolved through discussion until a consensus was reached.

### 2.5. Data Synthesis and Statistical Analysis

All the analyses were performed using the STATA SE 14.0 software (StataCorp, College Station, TX, USA). The time-to-event data were summarized as HR. Statistical heterogeneity among these studies was calculated by Cochran's Q test and *I*^2^ index (>50%, and *p* < 0.1 indicated high heterogeneity). The source of heterogeneity was investigated using subgroup analysis stratified by study design. The meta-analysis was performed using a random-effects model when significant heterogeneity (*p* < 0.1) was detected; otherwise, the fixed-effects model was adopted. A *p* value <0.05 indicated statistical significance. Next, we assessed the potential publication bias by visual inspection of the funnel plots. To assess whether a specific study had a dominant effect on the outcomes, individual studies were sequentially excluded, its effect on the overall estimate was evaluated, and Cochran's Q-test *p* value for heterogeneity was calculated.

## 3. Results

### 3.1. Study Selection

**Figure 1:**
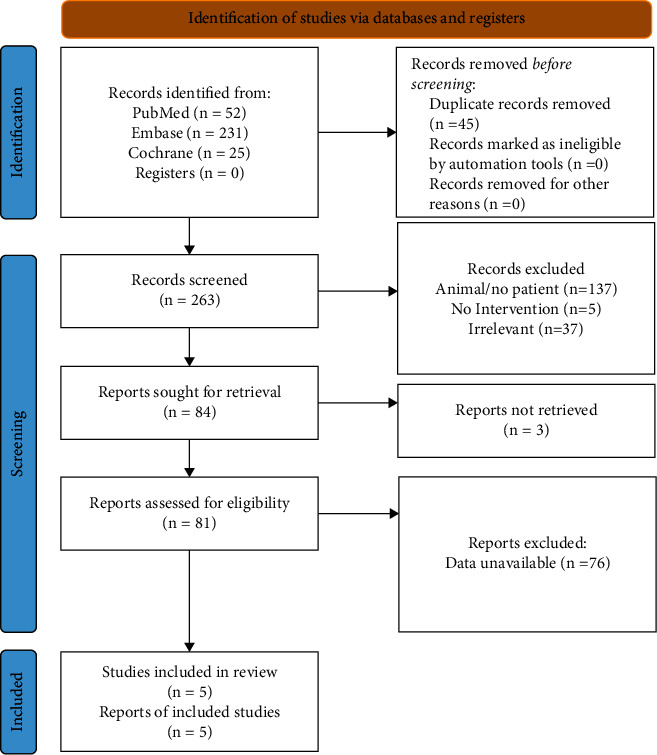
Flow diagram of the study selection process.

The systematic literature database retrieved 308 relevant documents. After initial screening, duplicate documents, reviews, conference abstracts, editorials, letters, notes/reports, surveys, and meta-analyses were removed. Two inaccessible documents and one non-English article were also excluded. The remaining 81 full-text articles or abstracts were assessed thoroughly for eligibility. Of these, 74 articles were excluded due to insufficient data on study design, population, intervention, and outcome. Subsequently, two animal-related articles were subsequently deleted. Finally, six eligible studies were included in the meta-analysis. The literature search and the selection flowchart are illustrated in Figure 1.

### 3.2. Characteristics of Included Studies

The characteristics of all included studies are summarized in Table 1. This meta-analysis consisted of six eligible studies including one RCT, four cohort studies, and one case-control, encompassing 5693 patients, including 1592 statin users and 4101 nonstatin users. Two studies were performed in Korea and UK, respectively, one in Spain and one in Taiwan. The mean or median age of statin-using patients varied from 53.5 to 72.5 years and 54.5 to 69.8 years in nonstatin users. The studies consisted of a higher proportion of males in nonstatin users compared to statin users. The follow-up time was 4–17 years. All patients underwent previous antitumor therapies, and the treatment modalities included surgery/chemotherapy/radiotherapy, surgery/chemotherapy, neoadjuvant therapy/gastrectomy/adjuvant therapy, or radical gastrectomy.

### 3.3. Quality Assessment

A low bias was noted in the RCT by Kim et al. due to the missing outcome data, measurement of the outcome, and selection of the reported results according to ROB 2.0 analysis [43]. Insufficient data were provided for the randomization process and deviations from intended interventions, and the overall bias of this study was unclear (Supplementary Table 1a). The overall qualities of four cohorts and one case-control studies were 6–8 on the NOS, suggesting low-to-moderate bias (Supplementary Tables 1b and 1c).

### 3.4. OS between Statins Users vs. Nonstatin Users

**Figure 2:**
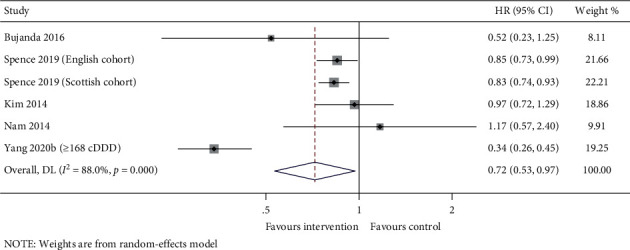
Forest plot of OS between statins users *vs*. nonstatin users.

The survival outcome between statins users and nonstatin users was compared in the six studies. The pooled analysis showed that statins added to standard treatment prolonged the patient's OS (HR (95% CI): 0.72 (0.53–0.97), *p* = 0.032). Also, significant heterogeneity was detected among the studies (*I*^2^ = 88.0%, *p*_*heterogeneity*_ < 0.001) (Figure 2).

### 3.5. Subgroup Analysis

**Figure 3:**
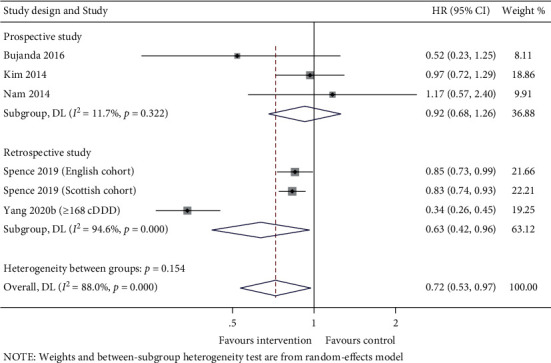
Forest plot of subgroup analysis by study design between statins users *vs*. nonstatin users.

This meta-analysis comprised three prospective and three retrospective studies. Subgroup analysis by study design did not find any significant difference in the OS between statins users and nonstatin users for the prospective studies (HR (95% CI): 0.92 (0.68–1.26), *p* = 0.614), whereas the retrospective studies suggested that the statins users might prolong the survival outcome (HR (95% CI): 0.63 (0.42–0.97), *p* = 0.032). Also, substantial heterogeneity was observed among the retrospective studies (*I*^2^ = 94.6%, *p*_*heterogeneity*_ < 0.001), but no heterogeneity was found among the prospective studies (*I*^2^ = 11.7%, *p*_*heterogeneity*_ = 0.322) (Figure 3).

### 3.6. Publication Bias

**Figure 4:**
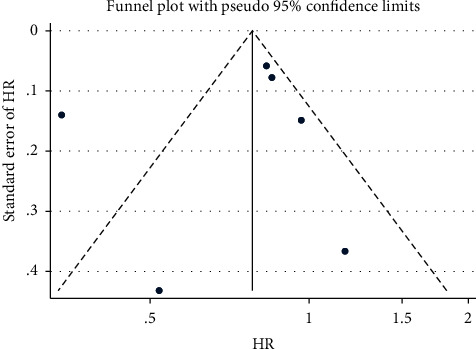
Funnel plot with pseudo 95% confidence limits.

Included studies were graphically assessed for any potential publication bias through a funnel plot. The studies were plotted with the estimated effect on the horizontal axis and the standard error of the estimated effect on the vertical axis. Studies with small samples scattered widely at the bottom of the graph, while large-sample studies were closer to the true effect of the intervention in the upper part of the plot. Studies with consistent estimate values were within the 95% CI. The funnel plot showed asymmetry, suggesting publication bias (Figure 4).

### 3.7. Sensitivity Analysis

**Figure 5:**
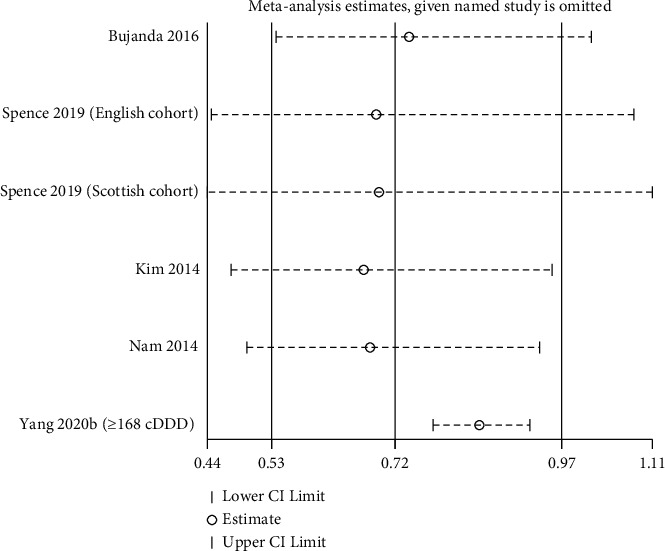
Sensitivity analysis for the OS between two groups.

Sensitivity analysis was performed by the sequential exclusion of the given-name study and re-evaluating the effects pooled from the remaining studies. The results demonstrated that our meta-analysis results were robust (Figure 5).

## 4. Discussion

GC is associated with a poor prognosis [2]. In recent years, significant efforts have been made to improve the prognosis of GC patients [45, 46]. Statins have been widely used for the treatment of lipid disorders [47, 48]. The potential anticancer effects of statins have been demonstrated in cultured tumor cells and animal tumor models [23, 25, 27]. Clinical evidence showed that statin therapy reduces the risk of GC and improves its prognosis [38, 44]. The current meta-analysis suggested that the use of statins on GC combined with standard treatment prolongs the patient's long-term OS outcome. In addition, subgroup analysis by study design indicated that the OS was significantly improved in statin users *vs*. nonstatin users in retrospective studies, whereas this survival benefit for statin users was not observed in prospective studies.

Various outcomes have been discussed with respect to the efficacy of statins in the treatment of GC. Previous studies focused only on the correlation between statins and the occurrence and development of GC. The large sample meta-analysis by Singh et al. [38], including 7 case-control, 1 cohort, and three post hoc analyses of 26 RCTs, showed that statins were modestly associated with reduced risk of GC in a dose-dependent manner. The summary estimate of the association between statins and GC was consistent after adjusting for the influence of specific confounding factors. However, long-term outcomes correlated with statin use were not discussed in the study. In addition, the results showed that the efficacy of statins was primarily noted in observational studies, whereas the RCTs did not demonstrate a significant preventive effect of statins against GC. The limitation of patient population selection and statistical power in the study design of RCTs was further explained.

The effect of statins on the prognosis of GC patients remained unclear. The RCT by Kim et al. in Korea suggested that simvastatin (40 mg daily) plus capecitabine-cisplatin (XP) therapy had no effect on the PFS and OS in patients with previously untreated advanced GC (AGC) [43]. Similarly, a 2016 Chinese study showed that simvastatin combined with XP chemotherapy did not prolong the PFS in patients with nonsurgical AGC [49]. Recently, several studies have been published on mortality outcomes. The study by Yang et al. in Taiwan suggested that statin use might improve the OS of patients with GC after surgery and adjuvant chemotherapy [44]. In two independent UK cohorts, statin use was associated with a moderately reduced cancer‐specific mortality [39]. Compared to a previous meta-analysis [38], the current study included six completely different latest observational studies and RCTs and analyzed the long-term OS outcome for statins users. The pooled results showed that statins might be associated with a survival benefit in GC patients.

In this meta-analysis, high heterogeneity was observed across the studies, which could be attributed to the patient's age, gender, tumor stage, previous antitumor treatment, statins type, dose and duration, follow-up time, and study design, location, and setting. However, subgroup analysis did not reveal any heterogeneity among the prospective studies, suggesting that study design might be a source of heterogeneity. Bujanda et al. performed the meta-regression analysis and indicated that the age of GC patients significantly modified the association between pravastatin therapy and cancer risk [50]. Additionally, Nam et al. showed that the overall use of statins did not improve the RFS or OS after the resection of stage II or III gastric cancer; however, the use of statins for >6 months could increase the survival rate [42].

Currently, there is no systemic review on the effect of statin use on GC patients' prognosis after standard treatments. This study consisted of both updated observational studies and RCTs and comprehensively analyzed the association between statins and OS. Furthermore, the sensitivity analysis suggested that our findings were robust.

Nevertheless, the present study has several limitations. First, this review inherited the limits of the included observational studies, and caution must be applied while extrapolating the results. Second, only one RCT was included, and the nonrandomized nature of this meta-analysis needs to be identified. Third, various statin formulations were prescribed to patients, but we were unable to analyze the OS associated with each statin type due to the limited number of included studies. Fourth, the data on tumor staging could not be identified in the retrospective studies, and confounding factors might occur as the individuals with a better prognosis (i.e., lower tumor stage) are more likely to receive statins than those with a poor prognosis. Fifth, patients using statins might not comply with the prescribed dosage. Finally, although many potential confounding factors were adjusted to estimate the association between statins and OS, various studies might use different variables, and specific potential confounders might not be included. We declare that this study was not registered, resulting in insufficient transparency.

## 5. Conclusions

Statins might improve the OS in GC patients, providing evidence for the antitumor effect of statins combined with surgery, radiotherapy, and chemotherapy in GC treatment. However, additional subgroup analysis based on the study design revealed that this improved survival outcome was only observed in retrospective, but not in prospective studies. Further prospective randomized studies are warranted to confirm the long-term benefits of statin use after standard treatment in patients with GC.

## Figures and Tables

**Table 1:** Literature search and characteristics of the included studies.

Study	Design	Country	Statin type	Sample size		Age (year, mean, or median)		Gender, male (%)		Previous therapy	Follow-up duration
				Statin users	Nonstatin users	Statin users	Nonstatin users	Statin users	Nonstatin users		
Kim et al. 2014	RCT	Korea	Simvastatin	120	124	53.5 (20–78)	54.5 (24–79)	91	85	Neoadjuvant therapy/gastrectomy/adjuvant therapy	5.4 years
Bujanda et al. 2016	Prospective cohort study	Spain	Pravastatin	20	40	65.4 (11.7)	66.3 (12.8)	15	23	Surgery/chemotherapy/radiotherapy	4–6 years
Spence et al. 2019 (English cohort)	Retrospective cohort study	UK	Statin prescriptions	650	1741	72.5 (8.9)	69.8 (12.8)	476	1141	Surgery/chemotherapy/radiotherapy	17 years
Spence et al. 2019 (Scottish cohort)	Retrospective cohort study	UK	Statin prescriptions	370	552	72.4 (9)	68.3 (13.1)	370	552	Surgery/chemotherapy/radiotherapy	17 years
Yang et al. 2020 (PSM)	Retrospective cohort study	Taiwan	Statin prescriptions	367	1468	64.3 (10.5)	64.1 (12.6)	208	857	Surgery/chemotherapy	14 years
Nam et al. 2014	Case-control	Korea	Statin prescriptions	65	176	—	—	18	42	Radical gastrectomy	3.5 years

## Data Availability

The datasets used and/or analyzed during the current study are available from the corresponding author on reasonable request.

## Supplementary Materials

Supplementary Materials Supplementary Table 1a: ROB 2.0 quality assessment for RCT. Supplementary Table 1b: NOS criteria quality assessment for cohort study. Supplementary Table 1c: NOS criteria quality assessment for case-control studies.
