# Supplementary material for: Statins Are Associated with Improved Survival of Patients with Gastric Cancer: A Systematic Review and Meta-Analysis
Source: Int J Clin Pract. 2022 May 17;2022:4938539. doi: 10.1155/2022/4938539 (PMC9158792; doi:10.1155/2022/4938539)
Supplement: Supplementary Materials — Supplementary Table 1a: ROB 2.0 quality assessment for RCT. Supplementary Table 1b: NOS criteria quality assessment for cohort study. Supplementary Table 1c: NOS criteria quality assessment for case-control studies. [file 4938539.f1.doc]

**Supplementary Table 1a.** ROB 2.0 quality assessment for RCT

| Study | Bias arising from the randomization process | Bias due to deviations from intended interventions | Bias due to missing outcome data | Bias in measurement of the outcome | Bias in selection of the reported result | Overall bias |
| --- | --- | --- | --- | --- | --- | --- |
| Kim 2014 | Unclear | Unclear | Low | Low | Low | Unclear |

*Low: low risk of bias; Unclear: unclear risk of bias.

**Supplementary Table 1b.** NOS criteria quality assessment for cohort study

| Study | Representativeness of the exposed cohort | Selection of the non-exposed cohort | Ascertainment of exposure | Demonstration that outcome of interest was not present at start of study | Comparability of cohorts on the basis of the design or analysis | Assessment of outcome | Was follow-up long enough for outcomes to occur | Adequacy of follow up of cohorts | Total quality scores |
| --- | --- | --- | --- | --- | --- | --- | --- | --- | --- |
| Bujanda 2016 | ⭐ | ⭐ | ⭐ | ⭐ | ⭐⭐ | ⭐ | / | / | 7 |
| Spence 2019 | ⭐ | ⭐ | ⭐ | / | ⭐⭐ | ⭐ | ⭐ | ⭐ | 8 |
| Yang 2020 | ⭐ | ⭐ | / | / | ⭐⭐ | ⭐ | ⭐ | ⭐ | 7 |

*A star was awarded for each high quality item, and a maximum of two stars could be given for Comparability. The highest quality studies were awarded up to nine stars (points) representing the least risk of bias.

**Supplementary Table 1c.** NOS criteria quality assessment for case-control studies

| Study | Is the case definition adequate? | Representativeness of the cases | Selection of controls | Definition of controls | Comparability of cases and controls on the basis of the design or analysis | Ascertainment of intervention | Same method of ascertainment for cases and controls | Non-response rate | Total quality scores |
| --- | --- | --- | --- | --- | --- | --- | --- | --- | --- |
| Nam 2014 | ⭐ | ⭐ | ⭐ | ⭐ | ⭐ | / | ⭐ | / | 6 |

*A star was awarded for each high quality item, and a maximum of two stars could be given for Comparability. The highest quality studies were awarded up to nine stars (points) representing the least risk of bias.
